# Synthesis and Antimicrobial Evaluation of Dibenzo[*b*,*e*]oxepin-11(6*H*)-one *O*-Benzoyloxime Derivatives

**DOI:** 10.3797/scipharm.1107-02

**Published:** 2011-09-18

**Authors:** Bassem Sadek, Carmen Limban, Camelia Elena Stecoza, Sigurd Elz

**Affiliations:** 1 Department of Pharmacology and Therapeutics, Faculty of Medicine and Health Sciences, United Arab Emirates University, P.O. Box 17666, Al-Ain, United Arab Emirates; 2 Pharmaceutical Chemistry Department, Faculty of Pharmacy, “Carol Davila” University of Medicine and Pharmacy, Traian Vuia 6, Sect. 2, 020956, Bucharest, Romania; 3 Department of Pharmaceutical/Medicinal Chemistry, Faculty of Chemistry and Pharmacy, University of Regensburg, University Str. 31, D-93053 Regensburg, Germany

**Keywords:** O-Acyloximes, Dibenzo[*b,e*]oxepine, Dibenzo[*b,e*]thiepine, Antimicrobial activity Antifungal activity, *E/Z* Isomerism of oximes (*cis/trans*)

## Abstract

A series of dibenzo[*b*,*e*]ox(thi)epin-11(6*H*)-one *O*-benzoyloximes has been synthesized and structurally elucidated by means of IR, ^1^H-NMR, ^13^C-NMR, MS, and elemental analysis. The newly developed compounds were screened at concentrations of 200–25 μg/mL for their antibacterial activity against Gram+ve organisms such as *Methicillin-Resistant Staphylococcus Aureus* (*MRSA*), Gram−ve organisms such as *Escherichia coli* (*E. coli*), and at the same concentration range for their antifungal activity against fungal strain *Aspergillus niger* (*A. niger*) by the cup plate method. Ofloxacin and ketoconazole (10 μg/mL) were used as reference standards for antibacterial and antifungal activity, respectively. The dibenzo[*b,e*]oxepines **6a–c** and **6e–h** showed low antimicrobial activity (MIC 125–200 μg/mL) compared to the reference substances, whereas a major improvement (MIC 50–75 μg/mL) was achieved with the synthesis of the corresponding bromomethyl derivative **6d**. Moreover, replacement of oxygen by its bioisosteric sulfur led to isomeric dibenzo[*b,e*]thi-epine derivatives **6g,h** which significantly exhibited higher antimicrobial activity (MIC 25–50 μg/mL) against all tested culture strains used in the present study, demonstrating that a change of chemical class from dibenzo[*b,e*]oxepine to dibenzo[*b,e*]thiepine significantly improves the antimicrobial activity. Further variation, such as the oxidation of the thiepine sulfur to the corresponding isomeric dibenzo[*b*,*e*]thiepine 5,5-dioxide derivative **9**, comparatively failed to exhibit high activity (MIC 200 μg/mL) against *S. aureus*, *E. coli* or *A. niger*.

## Introduction

The emergence of the antimicrobials resistance and multiresistance of bacterial and fungal infectious agents has urged the research for new antimicrobial substances and for new strategies for the treatment of infectious diseases which still remain a top public health problem in the world. The aim of this study was to evaluate the *in vitro* antimicrobial activity of some newly synthesized dibenzo[*b*,*e*]oxepin-11(6*H*)-one oxime derivatives which constitutes the fundamental structure of many products with biological activity including antidepressant [1–4], antipsychotic [5], antiinflammatory [6, 7], antibacterial and antifungal, and antidepressant activity [8]. Based on the facts that oximes and their derivatives have attracted considerable attention since the past few decades due to their chemotherapeutic value, as they were found to be antihyperglycemic [9], anti-neoplastic [10], anti-inflammatory [11], and antimicrobial [12], and in continuation of our investigations on the class of dibenzo[*b,e*]oxepines with potential pharmacological properties, the new series of isomeric dibenzo[*b*,*e*]oxepin-11(6*H*)-one *O*-benzoyloximes and dibenzo[*b*,*e*]thiepin-11(6*H*)-one *O*-benzoyloximes substituted with a methyl group at 2-position of dibenzo[*b,e*]ox(thi)epine nucleus has been synthesized and screened for the first time on antimicrobial activity [13].

## Results and Discussion

### Chemistry

The synthesis of title compounds was achieved in three stages.

#### First Stage: Synthesis of 2-(phenoxymethyl)benzoic acids (**3a–f**)

In the first stage, the substituted 2-(phenoxymethyl)benzoic acids (**3a–f**) were prepared by treating the phthalide (**1**) with correspondingly substituted potassium phenoxide (**2a–f**) in xylene. The resulted potassium salts of 2-(phenoxymethyl)benzoic acid or 2-[(4-methyl-phenoxy)methyl]benzoic acid showed a good solubility in an aqueous solution of 10% potassium hydroxide and were separated from xylene through precipitation upon acidification using a mineral acid solution. The potassium salts **2a–b** and of *p*-methylphenol **2c–f** were obtained using the corresponding phenol or *p*-methylphenol and potassium hydroxide in xylene, and the resulting water was removed by azeotropic distillation (Scheme 1).

#### Second Stage: Synthesis of dibenzo[*b*,*e*]oxepin-11(6*H*)-one (**4a–h**)

The intermediates **4a–f** were synthesized by a Friedel-Crafts cyclization of the corresponding (phenoxymethyl)benzoyl chloride or 4-[(4-methylphenoxy)methyl]benzoic acid in dry 1,2-dichloroethane. The acid chlorides were obtained by refluxing the **3a–f** with thionyl chloride in a 25 percentage excess and were instantly used in the next step without further purification (Scheme 1).

#### Third Stage: Synthesis of (E/Z)-dibenzo[*b*,*e*]oxepin-11(6*H*)-one *O*-benzoyloximes derivatives (**6a–f**)

Compounds **6a–f** (*E* and *Z*) were prepared by acylation of the correspondingly 2-sustituted oxime intermediates **5a–f** with different benzoyl chlorides in dry benzene under catalysis of anhydrous pyridine as a proton acceptor. The oxime intermediates **5a–f** were obtained by treating the ketones **4a–f** with hydroxylamine hydrochloride in the presence of pyridine. The reactions are presented in the Scheme 1 and the structures of the new compounds (**6a–h**) are presented in Table 1.

The synthesis of dibenzo[*b*,*e*]thiepin-11(6*H*)-one *O*-benzoyloximes **6g,h** and dibenzo[*b*,*e*]-thiepin-11(6*H*)-one 5,5-dioxide (sulfone) **9** was performed in several stages. In the first stage, the reaction of phthalide (**1**) with potassium salts of thiophenol **2g** or *p*-methyl-thiophenol **2h** resulted in 2-[(phenylthio)methyl]benzoic acid (**3g**) and 2-{[(4-methylphenyl)-thio]methyl}benzoic acid (**3h**), respectively. These acids were cyclized with polyphosphoric acid to the desired dibenzo[*b*,*e*]thiepin-11(6*H*)-one oxime **4g** and its corresponding 2-methyl derivate **4h**. In the second stage, the ketones **4g–h** were converted to the corresponding oxime intermediates **5g–h** upon treatment with hydroxylamine hydrochloride. The third stage comprised the acylation of oximes **5g**–**h** with various acid chlorides and afforded the new dibenzo[*b*,*e*]thiepin-11(6*H*)-one *O*-benzoyloximes **6g,h** (Scheme 2).

For synthesis of compound **9**, 2-methyldibenzo[*b,e*]thiepin-11(*6H*)-one (**4h**) and molybdenum trioxide were dissolved in ethanol, and upon addition of 30% aqueous hydrogenperoxide, the mixture was refluxed for 53 min. Water was added to the reaction mixture and the solid residue was filtered and recrystallized from ethanol to obtain the intermediate 2-methyldibenzo[*b*,*e*]thiepin-11(6*H*)-one 5,5-dioxide (**7**) which has been instantly converted to the oxime intermediate **8** by treatment with hydroxylamine hydrochloride in presence of anhydrous pyridine. 2-Methyldibenzo[*b*,*e*]thiepin-11(6*H*)-one *O*-benzoyloxime 5,5-dioxide (**9**) was prepared through an acylation reaction of **8** with benzoyl chloride under catalytic presence of anhydrous pyridine in absolute benzene (Scheme 3).

The structures **6a–h** and **9** were assigned to the isolated products on the basis of their elemental analyses and their high-field ^1^H- and ^13^C-NMR, IR, and mass spectral data. TLC, ^1^H and ^13^C NMR showed 2 isomers of oximes (*E*/*Z*). The ^1^H-NMR spectra of the new (*E*/*Z*)-dibenzo[*b,e*]ox(thi)epines are divided into two spectra, one corresponding to the ox(thi)epine system and another to the acyl radical attached to the oxime group. The presence of oxygen in 5-position favors the existence of *E*/*Z* isomerism which results in spectra with the dedoublation of the protons and the carbon signals. The protons of the methyl group situated in 2-position of dibenzo[*b,e*]ox(thi)epine nucleus give in the majority a broad singlet signal in the range of 2.48–2.29 ppm, and in theminority a singlet signal in the range 2.46–2.25 ppm. In addition, the protons of the methylene group (H-6) of the derivatives **6a–h** and **9** give a singlet signal in the range of 5.31–5.10 ppm, and a broad singlet signal in the range of 5.21–4.47 ppm, providing additional evidence for the existence of *E*/*Z* isomerism in titled compounds. Moreover, analysis of the ^13^C-NMR spectra of **6a**, **6e**, and **6g** indicated that the methylene group (C6) appears in majority (^M^) by 70.60 ppm and in minority (^m^) by 70.71, and the differences between the chemical shifts of the two *E*/*Z* isomers were found to be insignificant. Regarding the ^13^C-NMR analysis, the carbon atom C1 in the oxepine system is the most screened carbon atom, and the C11 is the most unscreened carbon atom and can be found in the range of 163.47–165.45. The signal corresponding to the C12 atom appears in the range of 161.5–164 ppm. The spectral data using ^1^H-NMR and ^13^C-NMR spectroscopy confirmed the structure of the obtained compounds as well as the existence of *E*/*Z* isomers.

### Biological Activity

The newly developed (*E*/*Z*)*-*dibenzo[*b*,*e*]ox(thi)epine derivatives were tested at concentrations of 200–25 μg/mL for their antibacterial activity against Gram+ve organisms such as methicillin-resistant *Staphylococcus aureus* (MRSA), Gram–ve organisms such as *Escherichia coli* (*E. coli*), and at the same concentration range for their antifungal activity against fungal strain *Aspergillus niger* (*A. niger*) by the cup plate method [14]. Ofloxacin and ketoconazole (10 μg/mL) were purchased from Wuhan Konglong Century Technology Development Co., Ltd. (Wuhan, China), and were used as reference standards for antibacterial and antifungal activity, respectively. The obtained in vitro antimicrobial results are listed in Table 1.

Despite the diversity of substituents at 2-position of the dibenzo[*b,e*]oxepine ring or the variety of *O*-benzoyl groups realized in derivatives **6a–c** and **6e,f**, low antimicrobial activity (MIC 125–200 μg/mL) compared to the reference substances Ofloxacin and Ketoconazole, was observed. Based on the investigation of integrals obtained for the protons (H-6) in the ^1^HNMR spectra of the derivatives **6a–c** and **6e,f**, a mixture of about 1:1.5 ratio was found and indicated the presence of one isomer (*E-* or *Z-*isomer) in majority, and might be the reason for the low antimicrobial activity observed. Major improvement in antimicrobial activity was obtained with the development of compound **6d**. Within this homogenous series, the *O*-benzoyl moiety bearing a bromomethyl function at *p*-position seemed to be optimal in exhibiting antimicrobial activity with MIC of 75 μg/mL against *E. coli* and *A. niger*, and a MIC of 50 μg/mL against *S. aureus*. A metabolic process within bacterial and fungal cells expected to be easier for **6d**, since an aromatic methylene group bearing a strong electron-withdrawing brome is present and might be an explanation for the increased antimicrobial activity obtained for **6d**, as the ration (*E*/*Z* or *Z*/*E*) was found to be similar to that of **6a–c** and **6e–f**. In contrast, the development of sulfur bioisosteres led to the dibenzo[*b*,*e*]thiepine derivatives **6g,h** which significantly exhibited higher antimicrobial activity (MIC 25–50 μg/mL) against all tested culture strains used in the present study, indicating that changes of the heteroatom at 5-positon of the dibenzo[*b,e*]oxepine ring obviously improves the antimicrobial activity. Moreover, the racemic mixture of 1:1 ratio obtained indicates that the quantity of one isomeric form (*E* or *Z*), which was found in minority for **6a–f**, is increased in **6g** and **6h**. The later finding signifies that the antimicrobial activity is also influenced by *E*/*Z*-isomerism and is assigned to only one specific isomer of titled compounds. On the other hand, an introduction of a sulfonyl moiety at 5-position resulted in compound **9** with 1:1 ratio (*E*/*Z*) which comparatively failed to exhibit antimicrobial activity (MIC 200 μg/mL) against *S. aureus*, *E. coli* or *A. niger*, demonstrating the negative impact of the sulfonyl group on the antimicrobial activity of titled compounds, apart from of the geometric isomerism present in compound **9** (Table 1).

## Experimental

### General procedures

Melting points are uncorrected and determined in open capillaries in a Buechi 512 Dr. Tottoli apparatus. ^1^H-NMR spectra were recorded on a Bruker WC 300 spectrometer with tetramethylsilane (TMS) as internal standard. Chemical shifts are reported in ppm downfield from internal tetramethylsilane as reference. ^1^H-NMR signals are reported in order: multiplicity (s…singlet; d…doublet; t…triplet; m…multiplet; *…exchangeable by D_2_O), number of protons, and approximate coupling constants in Hertz. For compounds **6a**, **6e**, and **6g** a ^13^C-NMR spectrum was recorded on a Bruker DPX 400 Avance (100 MHz) instrument and chemical shifts are reported in ppm downfield from internal tetramethylsilane used as reference. Elemental analyses were performed on Perkin-Elmer 240B and 240C instruments. Analyses (C, H, N) indicated by the symbols of elements were within ±0.4% of the theoretical values. Chromatographic separations were done using a Chromatotron Model 7924 (Harrison Research) with 4 mm layers of silica gel 60 PF containing gypsum (Merck). EI-mass spectra were recorded using Finnigan MAT CH7A (70 eV), Finnigan MAT 711 (80 eV), or Kratos MS 25 RF (70 eV) instruments. ^+^FAB-MS spectra were recorded on Finnigan MAT CH5DF instrument (xenon, DMSO)/glycerol).

### Chemistry

#### Synthesis of 2-(phenoxymethyl)benzoic acid (**3a,b**) and 2-[(4-methylphenoxy)methyl]-benzoic acid **3c–f**

A solution containing 0.05 mol of phenol for intermediates **3a** and **3b**, *p*-methylphenol for intermediates **3c–f** in 30 mL xylene was placed in a round-bottomed flask equipped with a Dean-Stark trap device. Subsequently, potassium hydroxide (0.055 mol) was added, and the reaction mixture was refluxed while the resulting water was removed by azeotropic distillation, and potassium salts **2a–f** were precipitated. Phthalide (**1**, 0.05 mol) was added and the mixture and refluxed until it solidifies. The precipitate was heated for solubilization with 10% potassium hydroxide solution and finally diluted with water (50 mL). The aqueous phase was separated and acidified with 1M hydrochloric acid solution until the mixture became acidic (pH 3), and benzoic acid intermediates **3a–f** were precipitated. The precipitate of each intermediate of **3a–f** was crystallized from a mixture of water/isopropanol (1:3) and shows a 49% yield. In the following step, each intermediate of **3a–f** (0.02 mol) was refluxed for three hours, and excess thionyl chloride together with the solvent were removed by reduced pressure, and the resulted benzoic acid chlorides in their crude status were used in the next step to prepare **4a–f**.

#### Synthesis of dibenzo[*b*,*e*]oxepin-11(6*H*)-ones **4a–f**

A suspension of benzoic acid chloride (0.02 mol) of each intermediate **3a–f** in 1,2-dichloro-ethane (25 mL), was added in portions to a stirring anhydrous aluminium chloride (0.02 mol) suspended in 1,2-dichloroethane (15 mL) which was maintained cooled at 0–5°C during the addition period. After the corresponding acid chloride was added, the reaction mixture was stirred at 5–20°C for one hour and then for another hour at 20°C. The mixture was then poured into 5% hydrochloric acid solution and stirred for one hour, the organic and aqueous layers were separated, washed once with 5% sodium hydroxide solution and twice with water, dried with anhydrous calcium chloride, treated with decolorizing charcoal, and evaporated under vacuum to yield the intermediates **4a–f** which were recrystallized from hexane in a 59% yield.

#### Synthesis of dibenzo[*b*,*e*]oxepin-11-(6*H*)-one *O*-benzoyloximes **6a–f**

For synthesis of target compounds **6a–f**, each of the precursors **4a–f** (0.05 mol) and hydroxylamine hydrochloride (0.15 mol) were boiled under reflux in pyridine (100 mL) for 96 h. The pyridine is subsequently distilled off in vacuum, and the resulted residue of **5a–f** was triturated with water, suction-filtered, dried and recrystallized from isopropanol in a 54% yield. In the following step, to a suspension of the corresponding intermediate **5a–f** (0.016 mol) in anhydrous benzene, a solution of correspondingly substituted benzoyl chloride (0.016 mol) in anhydrous benzene (10 mL) and dry pyridine (0.016 mol) was added dropwise and the mixture was refluxed for two hours. After cooling and filtration, the solvent was removed by distillation and the residue was triturated with isopropanol. The resulting solid was recrystallized from isopropanol to yield the title compounds **6a–f**.

#### Dibenzo[*b*,*e*]oxepin-11(6*H*)-one *O*-(4-iodobenzoyl)oxime (**6a**)

Yield: 73%, m.p.: 168.9–171.6 °C; IR (KBr): ν(cm^−1^) 2878 (CH_2_-O), 1739 (C=O oxime carbamate), 1585 (C=N); ^1^H-NMR (CDCl_3_): δ ppm = 7.77 (d, 2H, H-14, H-18, 8.6), 7.58 (d, 2H, H-15, H-17, 8.6), 7.46–7.51 (m, 4H, H-7, H-8, H-9, H-10), 7.37 (d, 1H, H-1, 3.1), 6.96 (dd, 1H, H-3, 9.0, 3.1), 6.84 (d, 1H, H-4, 8.9), 6.61 (dd, 1H, H-2, 6.61), 5.24, 5.20 (s, bs, 2H, H-6); ^13^C-NMR (CDCl_3_) δ ppm = 164.50 (C-11), 163.10 (C-12), 153.81 (C-4a), 137.90 (CH-15 and CH-17), 136.10 (C-3), 133.10 (C-10a), 131.0 (CH-14 and CH-18), 130.50 (C-8), 128.90 (C-2), 128.20 (CH-9), 128.10 (CH-13), 128.0 (C-7), 127.90 (C-10), 120.70 (C-4), 119.20 (C-1a), 113.0 (C-1), 101.30 (C-16), 70.71^m^ (C-6), 70.60^M^ (C-6), 20.74 (C-19); MS: m/z (%) 456 (M^+^, 10), 211 (13), 210 (100); Anal. Calcd. For C_21_H_14_INO_3_: C, 55.40; H, 3.10; N, 3.08. Found: C, 55.52; H, 3.34; N, 3.01.

#### Dibenzo[*b*,*e*]oxepin-11(6*H*)-one *O*-(3,4,5-trimethoxy benzoyl)oxime (**6b**)

Yield: 76%, m.p.: 182.4–185.1 °C; IR (KBr): ν(cm^−1^) 2837 (CH_2_-O), 1750 (C=O oxime carbamate), 1589 (C=N); ^1^H-NMR (CDCl_3_): δ ppm = 7.46–7.51 (m, 4H, H-7, H-8, H-9, H-10), 7.37 (d, 1H, H-1, 3.1), 7.01 (d, 2H, H-14, H-18, 8.6), 6.96 (dd, 1H, H-3, 9.0, 3.1), 6.84 (d, 1H, H-4, 8.9), 6.61 (dd, 1H, H-2, 6.61), 5.23, 5.19 (s, bs, 2H, H-6), 3.91, 3.89, 3.84, 3.80 (4*s, 9H, 4*OCH_3_); MS: m/z (%) 420 (M^+^, 11), 230 (29), 211 (13), 210 (100); Anal. Calcd. For C_24_H_21_NO_6_: C, 55.40; H, 3.10; N, 3.08. Found: C, 55.52; H, 3.34; N, 3.01.

#### 2-Methyldibenzo[*b*,*e*]oxepin-11(6*H*)-one *O*-(4-iodobenzoyl)oxime (**6c**)

Yield: 81%, m.p.: 156.4–159 °C; IR (KBr): ν(cm^−1^) 2912 (CH_2_-O), 1755 (C=O oxime carbamate), 1586 (C=N); ^1^H-NMR (CDCl_3_): δ ppm = 7.77 (d, 2H, H-14, H-18, 8.6), 7.62 (bs, 1H, H-1), 7.58 (d, 2H, H-15, H-17, 8.6), 7.46–7.51 (m, 4H, H-7, H-8, H-9, H-10), 7.08 (bdd, 1H, H-3, 8.1, 1.9), 7.00 (d, 1H, H-4, 8.1), 5.24, 5.20 (s, bs, 2H, H-6), 2.29, 2.26 (bs, s, 3H, CH_3_); MS: m/z (%) 470 (M^+^, 16), 225 (15), 224 (100); Anal. Calcd. For C_22_H_16_INO_3_ · ½ H_2_O: C, 55.23; H, 3.55; N, 2.93. Found: C, 55.63; H, 3.68; N, 2.83.

#### 2-Methyldibenzo[*b*,*e*]oxepin-11(6*H*)-one *O*-[4-(bromomethyl)benzoyl]oxime (**6d**)

Yield: 65%, m.p.: 189.1–190 °C; IR (KBr): ν(cm^−1^) 3029 (CH_2_-Br), 2925 (CH_2_-O), 1744 (C=O oxime carbamate), 1612 (C=N); ^1^H-NMR (CDCl_3_): δ ppm = 8.04 (d, 2H, H-14, H-18, 8.6), 7.85 (d, 2H, H-15, H-17), 7.62 (bs, 1H, H-1), 7.46–7.51 (m, 4H, H-7, H-8, H-9, H-10), 7.08 (bdd, 1H, H-3, 8.1, 1.9), 7.00 (d, 1H, H-4, 8.1), 5.23, 5.17 (s, bs, 2H, H-6), 4.62, 4.59 (bs, s, 2H, CH_2_Br), 2.34, 2.32 (bs, s, 3H, CH_3_); MS: m/z (%) 436 (M^+^, 12), 225 (15), 224 (100); Anal. Calcd. For C_23_H_18_BrNO_3_: C, 55.23; H, 3.45; N, 2.93. Found: C, 55.63; H, 3.68; N, 2.83.

#### 2-Methyldibenzo[*b*,*e*]oxepin-11(6*H*)-one *O*-(2-nitrobenzoyl)oxime (**6e**)

Yield: 81%, m.p.: 174.5–177.8 °C; IR (KBr): ν(cm^−1^) 2921 (CH_2_-O), 1757 (C=O oxime carbamate), 1596 (C=N); ^1^H-NMR (CDCl_3_): δ; ppm = 7.90 (d, 1H, H-15, 7.6), 7.61–7.53 (m, 3H, H-16, H-17, H-18), 7.36–7.19 (m, 4H, H-7, H-8, H-9, H-10), 7.14 (d, 1H, H-1, 3.2), 6.86 (dd, 1H, H-3, 8.9, 3.0), 6.73 (d, 1H, H-4, 9.0), 5.10, 5.02 (s, bs, 2H, H-6), 2.31, 2.25 (bs, s, 3H, CH_3_); ^13^C-NMR (CDCl_3_) δ; ppm = 165.45 (C-11), 161.50 (C-12), 153.93 (C-4a), 148.30 (CH-15), 130.87 (C-9), 129.89 (CH-17), 128.94 (C-2), 128.65 (C-7), 128.57 (C-8), 127.94 (C-10), 127.80 (C-13), 127.75 (CH-16), 124.60 (CH-14), 121.21 (C-4), 120.90 (C-3), 119.08 (C-1a), 113.14 (C-1), 70.71^m^ (C-6), 70.60^M^ (C-6); MS: m/z (%) 489 (M^+^, 25), 240 (11), 225 (15), 224 (100); Anal. Calcd. For C_22_H_16_N_2_O_5_ · ½ H_2_O: C, 66.48; H, 4.28; N, 7.05. Found: C, 66.84; H, 4.37; N, 6.86.

#### 2-Methyldibenzo[*b*,*e*]oxepin-11(6*H*)-one *O*-(3,4,5-trimethoxy benzoyl)oxime (**6f**)

Yield: 79%, m.p.: 185.3–187.1 °C; IR (KBr): ν(cm^−1^) 2938 (CH_2_-O), 1753 (C=O oxime carbamate), 1591 (C=N); ^1^H-NMR (CDCl_3_): δ; ppm = 7. 46–7.51 (m, 4H, H-7, H-8, H-9, H-10), 7.14 (d, 1H, H-1, 3.2), 7.01 (d, 2H, H-14, H-18, 8.6), 6.86 (dd, 1H, H-3, 8.9, 3.0), 6.73 (d, 1H, H-4, 9.0), 5.23, 5.19 (s, bs, 2H, H-6), 3.91, 3.89, 3.84, 3.80 (4*s, 9H, 4*OCH_3_), 2.31, 2.29 (bs, s, 3H, CH_3_); MS: m/z (%) 434 (M^+^, 17), 230 (46), 225 (14), 224 (100); Anal. Calcd. For C_25_H_23_NO_6_: C, 67.85; H, 5.43; N, 3.17. Found: C, 68.06; H, 5.23; N, 3.05.

#### 2-[(Phenylthio)methyl]benzoic acid (**3g**)

For synthesis of **3g**, 0.1 mol potassium hydroxide was added to a solution of 0.1 mol thiophenol in 60 mL xylene and refluxed until 2 mL of water were removed. Upon addition of 0.1 mol phthalide, the mixture was refluxed for 3h, cooled, and the solidified mixture was dissolved in 10% potassium hydroxide and diluted with 100 mL water. The aqueous phase was separated and acidified 1M hydrochloric acid (pH= 3) to afford **3g** which was filtered and recrystallized from aqueous ethanol.

#### 2-[4-Tolylthio)methyl]benzoic acid (2-{[(4-Methylphenyl)sulfanyl]methyl}benzoic acid, **3h**)

Similarly to the synthesis of **3g**, 2-[4-tolylthio)methyl]benzoic acid (**3h**) was achieved through the reaction of 0.1 mol *p*-(methyl)thiophenol and 0.1 mol phthalide, and re-crystallization from aqueous ethanol.

#### Dibenzo[*b*,*e*]thiepin-11(6*H*)-one (**4g**)

140 g polyphosphoric acid was heated to 80°C and 0.1 mol of 2-[(Phenylthio)methyl]-benzoic acid **3g** were slowly added under stirring, and the mixture was heated for one hour to 100–110°C. After partial cooling (80°C), ice and water were added, product **4g** was extracted with dichloromethane and washed with water and 5% sodium hydroxide. The solvent was removed under vacuum and the residue recrystallized from isopropanol and used in the next step without further characterization.

#### 2-Methyldibenzo[*b*,*e*]thiepin-11(6*H*)-one (**4h**)

Cyclodehydration of 2-[4-tolylthio)methyl]benzoic acid (**3h**; 0.1 mol) in the presence of polyphosphoric acid, by heating for 2.5 h to 140–150°C was carried out similarly to the procedure of **4g**; the crude product **4h** was recrystallized from ethanol, and used in the following step without further characterization.

#### Dibenzo[*b*,*e*]thiepin-11(6*H*)-one oximes **5g** and **5h**

For the synthesis of **5g** and **5h**, a mixture of 0.05 mol of corresponding dibenzo[*b*,*e*]-thiepin-11(6*H*)-one (**4g**) and 2-methyldibenzo[*b*,*e*]thiepin-11(6*H*)-one (**4h**) was refluxed for 24h with 0.15 mol hydroxylamine hydrochloride in 100 mL of pyridine. The pyridine was subsequently removed under vacuum, the residue of the corresponding product **5g** or **5h** was triturated with water and filtered, dried and finally recrystallized from isopropanol. Bothe oxime intermediates **5g** and **5h** were used instantly in the next step of oxime acylation to afford the final compounds **6g** and **6h** in similarity to the procedure carried out for compounds **6a–f**.

#### Dibenzo[*b*,*e*]thiepin-11(6*H*)-one *O*-(4-chlorobenzoyl)oxime (**6g**)

Yield: 83%, m.p.: 141.7–143 °C; IR (KBr): ν(cm^−1^) 2963 (CH_2_-S), 1757 (C=O oxime carbamate), 1590 (C=N); ^1^H-NMR (CDCl_3_): δ; ppm = 7.77 (d, 2H, H-14, H-18, 8.7), 7.58 (d, 2H, H-15, H-17, 8.7), 7.46–7.51 (m, 4H, H-7, H-8, H-9, H-10), 7.37 (d, 1H, H-1, 3.1), 6.96 (dd, 1H, H-3, 9.0, 3.1), 6.84 (d, 1H, H-4, 8.9), 6.61 (dd, 1H, H-2, 6.61), 4.66, 4.20 (s, bs, 2H, H-6); ^13^C-NMR (CDCl_3_) δ; ppm = 165.45 (C-11), 161.50 (C-12), 153.93 (C-4a), 148.30 (CH-15), 131.80 (CH-18), 130.87 (C-9), 129.89 (CH-17), 128.94 (C-2), 128.65 (C-7), 128.57 (C-8), 127.94 (C-10), 127.80 (CH-13), 127.75 (CH-16), 124.60 (CH-14), 121.21 (C-4), 120.90 (C-3), 119.08 (C-1a), 113.14 (C-1), 70.71^m^ (C-6), 70.60^M^ (C-6), 20.75 (C-19); MS: m/z (%) 380 (M^+^, 11), 227 (14), 226 (100), 185 (30); Anal. Calcd. For C_21_H_14_NO_2_SCl: C, 66.40; H, 3.71; N, 3.69. Found: C, 66.55; H, 3.72; N, 3.62.

#### 2-Methyldibenzo[*b*,*e*]thiepin-11(6*H*)-one *O*-(4-bromobenzoyl)oxime (**6h**)

Yield: 79%, m.p.: 205–207 °C; IR (KBr): ν(cm^−1^) 2966 (CH_2_-S), 1751 (C=O oxime carbamate), 1591 (C=N); ^1^H-NMR (CDCl_3_): δ; ppm = 7.77 (d, 2H, H-14, H-18, 8.6), 7.62 (bs, 1H, H-1), 7.58 (d, 2H, H-15, H-17, 8.6), 7.46–7.51 (m, 4H, H-7, H-8, H-9, H-10), 7.08 (bdd, 1H, H-3, 8.1, 1.9), 7.01 (d, 1H, H-4, 8.1), 4.66, 4.20 (s, bs, 2H, H-6), 2.29, 2.26 (bs, s, 3H, CH_3_); MS: m/z (%) 439 (M^+^, 44), 437 (23), 241 (17), 240 (100); Anal. Calcd. For C_22_H_16_NO_2_SBr: C, 60.28; H, 3.68; N, 3.20. Found: C, 60.03; H, 3.35; N, 3.14.

#### 2-Methyldibenzo[*b*,*e*]thiepin-11(6*H*)-one *O*-benzoyloxime 5,5-dioxide (**9**)

To a mixture of 2-methyldibenzo[*b,e*]thioepin-11(6*H*)-one (**4h**, 1 mmol) and MoO_3_ (0.05 mmol, 0.007g) in EtOH (2ml), 30% aq. H_2_O_2_ (0.3 ml, 2.67 mmol) was added and the mixture was refluxed for 53 min. After completion of the reaction, water (15 mL) was added and the reaction mixture was filtered. The solid residue was recrystallized from ethanol to obtain a pure product of 2-methyldibenzo[*b*,*e*]thiepin-11(6*H*)-one 5,5-dioxide (**7**) in 97% yield. The oxime intermediate **8** was prepared through treatment of **7** (1 mmol) with hydroxylamine hydrochloride (2 mmol) in presence of anhydrous pyridine. The desired product **9** was achieved through an acylation reaction of 2-methyldibenzo[*b*,*e*]thiepin-11(6*H*)-one oxime 5,5-dioxide (**8**, 1 mmol) with benzoyl chloride (1 mmol) in absolute benzene, and under catalytic presence of anhydrous pyridine.

Yield: 65%, m.p.: 183.5–185 °C; IR (KBr): ν(cm^−1^) 2926 (CH_2_-SO_2_), 1758 (C=O oxime carbamate), 1560 (C=N); ^1^H-NMR (CDCl_3_): δ; ppm = 7.72–7.62 (m, 6H, H-1, H-14, H-15, H-16, H-17, H-18), 7.46–7.51 (m, 4H, H-7, H-8, H-9, H-10), 7.08 (bdd, 1H, H-3, 8.1, 1.9), 7.01 (d, 1H, H-4, 8.1), 5.31, 4.47 (bs, bs, 2H, H-6), 2.48, 2.46 (bs, s, 3H, CH_3_); MS: m/z (%) 392 (M^+^, 4), 275 (11), 260 (10), 258 (100); Anal. Calcd. For C_22_H_17_NO_4_S: C, 67.52; H, 4.35; N, 3.80. Found: C, 67.58; H, 4.40; N, 3.44.

### Antimicrobial Activity

The quantitative *in vitro* antimicrobial study was carried on Muller-Hinton agar (Hi-media) plates (37 °C, 24 h) by the agar diffusion cup plate method [14]. The compounds (200–25 μg/mL) were screened for antimicrobial activity against the bacterial strains *Staphylococcus aureus* ATCC 25923 (*S. aureus*) (Gram+ve) and *Escherchia coli* ATCC 35218 (*E. coli*) (Gram−ve). Antifungal activity was tested on Sabouraud dextrose agar (Hi-media) plates (26 °C, 48–72 h) by the cup plate method against *Aspergillus niger* A733 (*A. niger*) also at a concentration level of 200–25 μg/mL. Ofloxacin and ketoconazole were used as standards for comparison of antibacterial and antifungal activity under the similar conditions. DMF was used as a solvent control for both antibacterial and antifungal activities, and the results are presented in minimal inhibition concentration (MIC) values (μg/mL) in Table 1.

## Conclusion

The new *E*/*Z*-compounds **6a**–**h** and **9** clearly differ in their corresponding antimicrobial activity depending on the type of substitution and that of the geometric ratio obtained for the titled compounds. Among the dibenzo[*b,e*]oxepines **6a–f** (ratio of *E*/*Z* or *Z*/*E* 1:1.5) developed in the course of this study, particularly **6d** which is possessing a bromomethyl substitution at *p*-position of *O*-benzoyloxime moiety was identified as exhibiting high antibacterial activity against *methicillin-resistant S. aureus* (Gram positive) and *E. coli* (Gram negative) bacteria and antifungal activity against *A. niger*. However, the isomeric dibenzo[*b,e*]thiepine derivatives **6g** and **6h** (ratio of *E*/*Z* 1:1) were found to be highest in their antibacterial activity. On the other hand, an introduction of a sulfonyl moiety at 5-position resulted in compound **9** with 1:1 ratio (*E*/*Z*) which, despite the geometric isomerism present, comparatively failed to exhibit antimicrobial activity (MIC 200 μg/mL) against *S. aureus*, *E. coli* or *A. niger*, demonstrating the negative impact of sulfonyl group on the antimicrobial activity of titled compounds. These distinct in vitro antimicrobial results, combined with the potential benefits or at least differences in geometric isomerism and pharmacokinetics, make the titled (*E*/Z)-dibenzo[*b*,*e*]ox(thi)epin-11(6*H*)-one *O*-benzoyloxime derivatives not only interesting leads for the further chemical geometrical separation of *E*- and *Z*–isomers within this series but also potentially interesting for additional structure-activity relationship studies.

## Figures and Tables

**Sch. 1:**
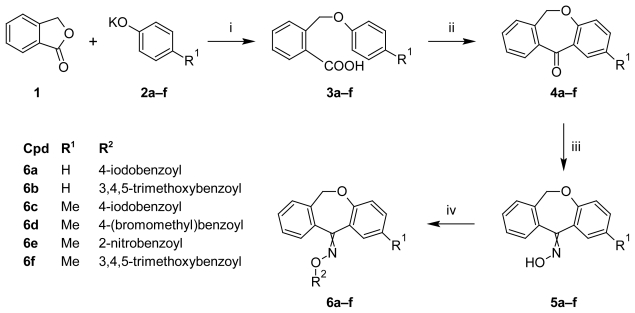
Synthesis of compounds **6a–f**. i: xylene, reflux, 5 h, 1 N NaOH, 1 M HCl; ii: a) SOCl_2_, reflux, 3 h; b) AlCl_3_, 0–5°C; c) stirring, 5–20°C, 1 h; iii: Pyridine, NH_2_OH·HCl, reflux, 96 h; iv: anhyd. benzene, pyridine, corresp. substituted benzoylchloride, reflux 2 h.

**Sch. 2:**
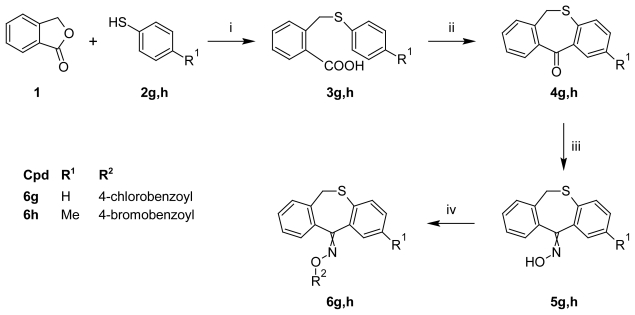
Synthesis of compounds **6g,h**. i: xylene, reflux, 5 h, 1N NaOH, 1 M HCl; ii: a) polyphorphoric acid, 80°C during addition of **3g** or **3h**; b) for intermediate **4g**: 100–110°C, 1h, and for intermediate **4h**: 140–150°C, 2.5 h; c) 80°C, ice-water, 1 N NaOH; iii: pyridine, NH_2_OH·HCl, reflux, 24 h; iv: anhydr. benzene, pyridine, corresp. substituted benzoylchloride, reflux 2 h.

**Sch. 3:**
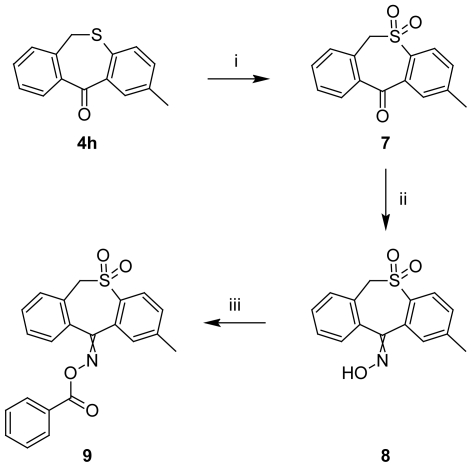
Synthesis of compound **9.** i: MoO_3_, ethanol, 30% H_2_O_2_, reflux, 53 minutes; ii: pyridine, NH_2_OH·HCl, reflux, 24 h; iv: anhydr. benzene, pyridine, benzoylchloride, reflux 2 h.

**Tab. 1:** *In vitro* antimicrobial activity of the title compounds

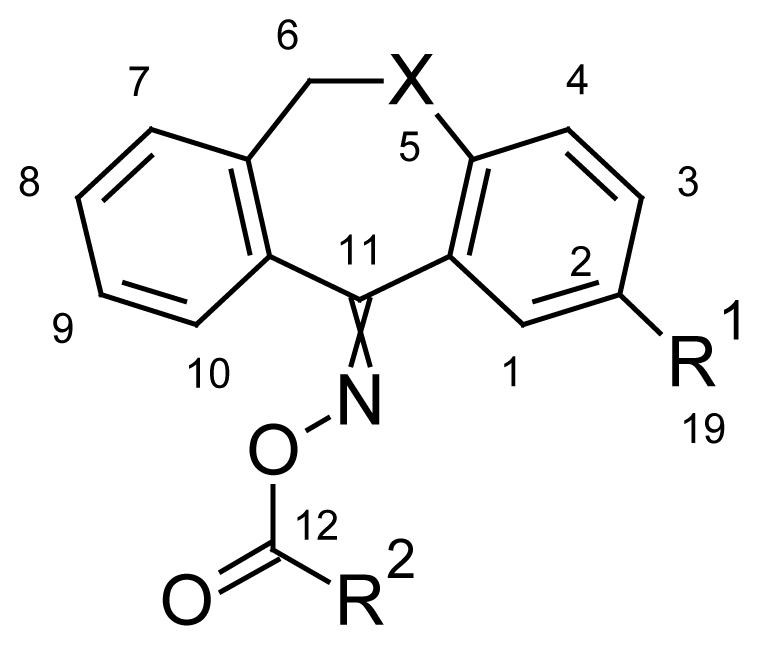						
				MIC μg/mL		
Compound	X	R^1^	R^2^	*S. aureus*	*E. coli*	*A. niger*
**6a**	O	H	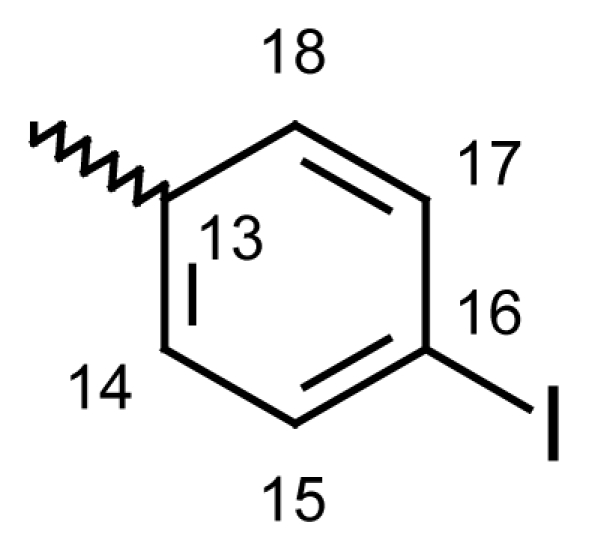	200	200	200
**6b**	O	H	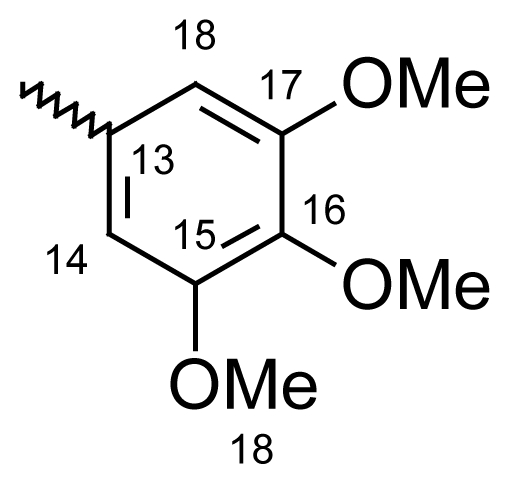	150	200	150
**6c**	O	CH_3_	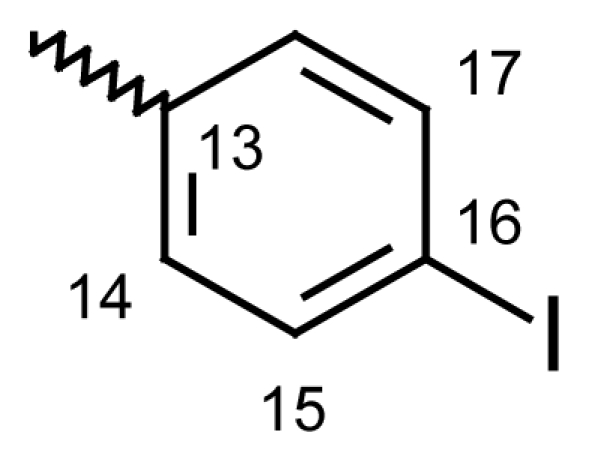	125	150	125
**6d**	O	CH_3_	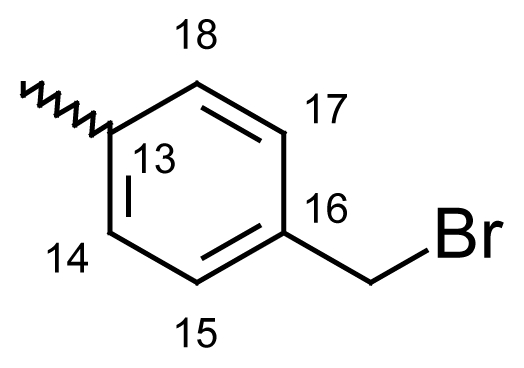	75	75	50
**6e**	O	CH_3_	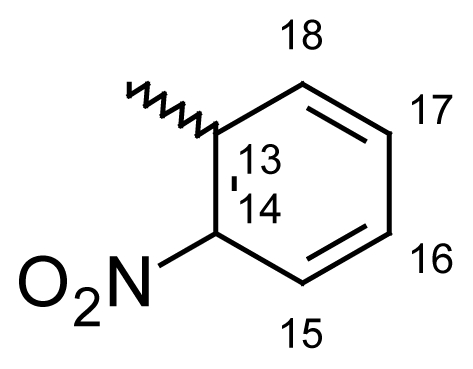	125	125	150
**6f**	O	CH_3_	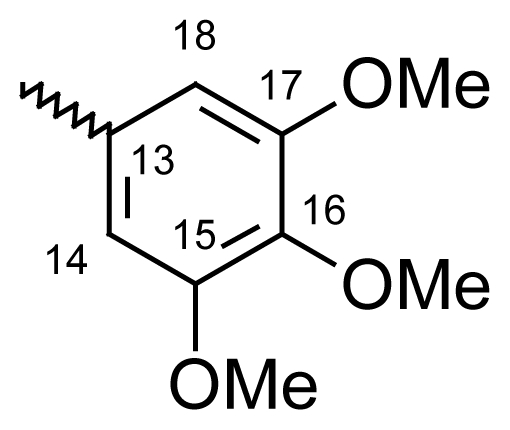	200	200	200
**6g**	S	H	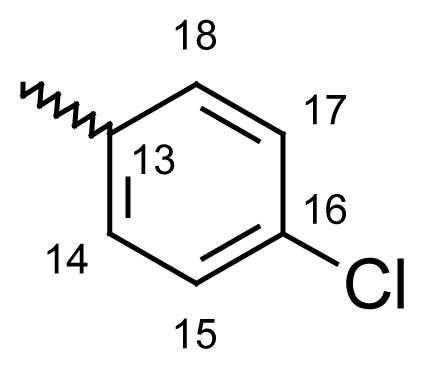	50	25	25
**6h**	S	H	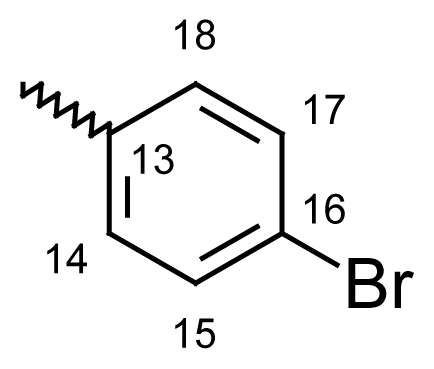	50	50	25
**9**	SO_2_	CH_3_	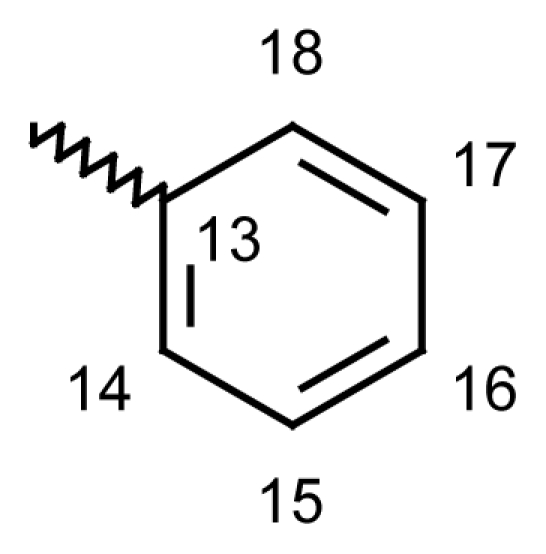	200	200	200
**Ofloxacin**	**–**	**–**	**–**	10	12.5	–
**Ketoconazole**	**–**	**–**	**–**	–	–	12.5
